# Intratendon bioimpedance spectroscopy: a first step exploring reliability and sensitivity to change

**DOI:** 10.7717/peerj.21084

**Published:** 2026-05-21

**Authors:** Patricia Beltrá, Miguel Delicado Miralles, Emilio Tomás-Muñoz, Rodrigo Martín-San Agustín, Adrian Escriche-Escuder, Enrique Velasco

**Affiliations:** 1 Neuroscience in Physiotherapy (NiP), Independent Research Group, Elche, Alacant, Spain; 2 Department of Physiotherapy, Universidad de Valencia, Valencia, Spain; 3 Universidad Europea de Valencia, Faculty of Health Sciences, Department of Nursing, Valencia, Spain; 4 Center for Translational Research in Physical Therapy (CEIT), Department of Pathology and Surgery, Faculty of Medicine, University Miguel Hernández, Alicante, Spain; 5 Department of Physiotherapy, University of Valencia, Clinimetry and Technological Development in Therapeutic Exercise Research Group (CLIDET), Valencia, Spain; 6 Laboratory of Ion Channel Research, Department of Cellular and Molecular Medicine, KU Leuven, Belgium

**Keywords:** Bioimpedance spectroscopy, Patellar tendon, Reliability, Sensitivity to change, Electrical characterization, Intratendon bioimpedance, Healthy volunteers

## Abstract

**Background:**

Tendon structure evaluation is fundamental for research and clinical practice, with the ultrasonography being the primary macrostructural imaging tool. Nevertheless, microstructural tendon adaptations require highly invasive procedures to be evaluated, *i.e.*, tendon biopsies. To address this, the present study aims to explore the reliability and sensitivity to change of a minimally invasive Intra-Tendon Bioimpedance Spectroscopy (IT-BIS), which measures the tendon’s electrical response across frequencies using fine needles inserted longitudinally along the tendon.

**Methods:**

Forty healthy volunteers were placed in the supine position with the knee flexed at 30°. Four needles were inserted under ultrasound guidance along the patellar tendon and connected to the IT-BIS device in a tetrapolar configuration for impedance measurement. IT-BIS was assessed twice per session and there were two sessions separated by at least two weeks. To evaluate intra- and inter-session reliability we calculated indexes of relative (Intra-class Correlation Coefficient: ICC_1,1_) and absolute reliability (Standard Error of Measurement: SEM. Minimal Detectable Change: MDC). Resistance, reactance, magnitude, and phase angle were measured across frequencies to characterize the tendon architecture. The high and low frecuency cutoff was established by the experimental characteristic frequency of our data at basal conditions. Also, intra-tendon electrolysis was applied in the second session to determine sensitivity to change of the IT-BIS.

**Results:**

IT-BIS showed excellent intrasession reliability across all parameters in both high and low frequency ranges with ICCs of 0.927 to 0.999. However, inter-session reliability was predominant poor (ICCs 0.087 to 0.390), with moderate reliability in phase angle at low frequency (ICC = 0.528). Sensitivity to change by needle insertion was evidenced at low frequency in all parameters, specifically resistance (*p* = 0.009; SRM = −0.54), reactance (*p* = 0.004; SRM −0.61), magnitude (*p* = 0.039; SRM = −0.4), and phase angle (*p* = 0.02; SRM = −0.67). However, sensitivy to change by percutaneous electrolysis was at high frequency, being resistance (*p* = 0.002; SRM = 0.68), reactance (*p* = 0.037; SRM = 0.39), magnitude (*p* = 0.01; SRM = 0.55), and phase angle (*p* = 0.006; SRM = 0.57). Minimal adverse effects were reported: 4.63% pain occurrence rate and were consistently mild and transient.

**Conclusion:**

Minimally invasive IT-BIS showed excellent intra-session reliability but poor-to-moderate inter-session reliability, underscoring the need for further methodological standardization. The technique demonstrated sensitivity to acute physiological changes, identifying needle insertion effects at low frequencies and percutaneous electrolysis effects at high frequencies. Adverse effects were minimal and mild. Overall, IT-BIS emerges as a safe and promising tool for tendon assessment.

## Introduction

Structural evaluation is a crucial part of tendon assessment in both clinical and research contexts (Malliaras et al., 2012; Millar et al., 2021). Imaging is the most common approach, with modalities such as quantitative ultrasound or magnetic resonance imaging (Sugrañes et al., 2023). Recently, other techniques have gained relevance, including elastrography (Mifsud et al., 2023), which quantifies tissue stiffness through different modalities (strain, shear wave, continuous shear wave and 3D elastography). Advanced magnetic resonance imaging (MRI) techniques, such as quantitative ultrashort echo time–T2* (UTE-T2*), which uses short and ultrashort echo times to enhance spatial resolution (Xie et al., 2020), and ultrasound tissue characterization (Van Ark et al., 2019), also offering valuable insights into tissue structure. Moreover, the correlation between imaging findings and clinical presentation of tendon pathologies, such as tendinopathy, remains weak (Docking, Ooi & Connell, 2015; De Jonge et al., 2015). One proposed explanation is that the clinical manifestation of tendinopathy (*i.e.,* painful tendon) may be the result of years of pathological progression (Fredberg & Stengaard-Pedersen, 2008). Consequently, it would be of interest for clinicians and researchers to develop tools that can evaluate new tissue properties potentially associated with clinical status and prognosis, as well as identify disease-related changes at earlier stages. Tendon biopsies and biomarker identification align with this approach, but there is still insufficient evidence to associate them with the diagnosis and prognosis (Zhu et al., 2021). The invasiveness and complexity of these methods also hinders their application.

Bioimpedance is a technique that measures the opposition of biological tissue to alternating electrical current, expressed as impedance, which combines resistance and reactance, both inversely related to tissue conductivity (Scott et al., 2020). Bioimpedance offers a valuable approach to examining the electrical properties of tissues, particularly by analyzing the composition of intracellular and extracellular water, reflecting changes in structure of different microscopic components of the sample (Jaffrin & Morel, 2008). In addition, bioimpedance systems are cost-effective, portable, and technically suitable for embedded or point-of-care implementations, which distinguishes them from the previous complex imaging-based techniques. Commonly, it has been used to estimate body water and composition, however, more localized and innovative applications have emerged, including the identification of muscle injuries (Nescolarde et al., 2023) and the guidance of lumbar puncture procedures (Sievänen et al., 2021). Regarding tendon pathology, animal studies have demonstrated that bioimpedance can detect a collagenase-induced tendon injury (Yoon et al., 2010; Ye et al., 2022). Nevertheless, studies investigating the use of tendon bioimpedance in humans have not been reported yet.

Taken together, current assessment techniques provide valuable structural and mechanical insights, yet they often show poor correspondence with clinical symptomatology in tendinopathy. Furthermore, they do not reflect microstructural changes in the tendon tissue. This gap highlights the need for minimally invasive, composition-sensitive tools to complement conventional imaging in the evaluation of tendon health.

Here we propose, for the first time, Intra-Tendon Bioimpedance Spectroscopy (IT-BIS) as a novel, minimally invasive, assessment tool usable in humans, with the capability of measuring potential microstructural changes in the tendon. Specifically, the aim of this study was to explore the reliability of the IT-BIS for electrical characterization of patellar tendon in healthy subjects. Also, we aimed to conduct a preliminary exploration of the sensitivity to change of the IT-BIS using an external stimulus to alter the tendon structure: a single session of Percutaneous Electrolysis (PE), an alternative treatment used for tendinopathies (Canosa-Carro et al., 2022; Ferreira, Araujo & De-La-Cruz-Torres, 2024) that has been documented to produce changes in the molecular microenvironment of healthy and pathological tendons (Sánchez-Sánchez et al., 2020; Peñin Franch et al., 2022). Finally, we also aimed to explore the potential adverse effects produced during the measurement, to evaluate whether the IT-BIS, a minimally invasive procedure, is safe for assessing tendon structure.

## Materials & Methods

### Study design

To assess the intra- and inter-session reliability of IT-BIS for the electrical characterization of the patellar tendon and its sensitivity to detect changes induced by PE, an observational study was conducted, as recommended in the COnsensus-based Standards for the selection of health Measurement INstruments for this type of study (Mokkink et al., 2010). For this purpose, bioimpedance measurements were performed in two sessions separated by at least two weeks. Intra-session reliability was examined during the first session by performing two measurements separated by 5 min. Inter-session reliability was assessed by comparing the measurements of the first session with an additional measure performed in a second session following the same protocol.

**Figure 1:**
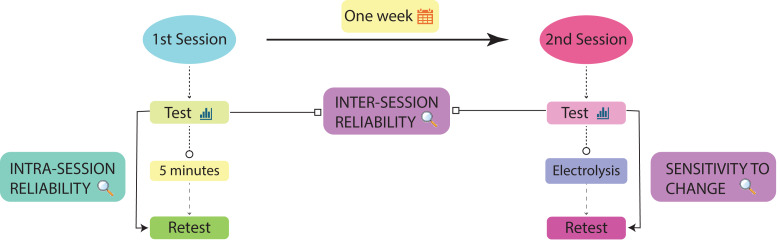
Flowchart representing the experimental design procedure.

Afterwards, to explore the sensitivity of bioimpedance to detect tendon changes, both solely due to the presence of an external body such as the needle and the combination of the needle with the effects of PE, a PE technique was applied to induce both mechanical and electrochemical in the patellar tendon. This technique involves inserting a needle and applying a galvanic current of 3 mA to generate a controlled microlesion and produce a non-thermal electrochemical effect. This reaction results in the formation of sodium hydroxide and localized changes in pH, as well as an increase in oxygen concentration at the treated site (Augustyn & Paez, 2022). Thus, a bioimpedance measurement was taken with only the PE needle placed intratendon, and another bioimpedance measurement was taken post-PE to determine whether these changes could be detected (Fig. 1).

The examiner was a physical therapist with more than five years of experience in invasive procedures in the field of physical therapy, such as PE, ensuring procedural consistency. The examiner was not blinded, the use of objective impedance recording and standardized electrode positioning procedures were intended to minimize potential operator-related bias. All measurements were performed under controlled conditions, including the same laboratory setting and ambient temperature across both sessions. It is important to control the ambient temperature because it can affect the impedance values. Specifically, impedance measured from the skin, increases at low temperatures (15 °C) and decreases at higher temperatures (35 °C) (Buono et al., 2004). Probably the IT-BIS could be less affected by temperature because is assessed percutaneously, but it was controlled as a precaution.

### Participants

Forty healthy volunteers were evaluated, from Elche, Spain. The specific inclusion criteria were: (1) age between 18 and 45 years; (2) no history of knee surgery in the last 2 years; and (3) absence of pain episodes in the lower limb within 30 days prior to data collection. The exclusion criteria were: (1) people with any illness/condition contraindicating current or needle application, such as coagulation deficits, allergies or belonephobia; (2) any severe conditions such as diabetes mellitus, cancer, neurological disease, depression, fibromyalgia and immunodepression; (3) being pregnant; (4) being a professional athlete; and (5) to obtain less than 88 points in the VISA-P questionnaire (Mendonça et al., 2016).

Participants were instructed to comply with the following guidelines before data collection: (a) avoid engaging in strenuous exercise for 48 h before the test, (b) not to consume anticoagulants, antidepressants, gabapentinoids or opioids one week before measurements, and (c) not to consume caffeine or energy drinks for at least 2 h before test. Before initiating the measurements, the evaluator asked the subjects to confirm that these guidelines had been followed and the VISA-P questionnaire was completed for each subject to ensure that the patellar tendon was in an adequate healthy state (Hernandez-Sanchez, Hidalgo & Gomez, 2011). All participants received a detailed explanation of the study procedures and signed informed consent. The study was approved by the Ethical Committee of Pharmacological Research in the University General Hospital of Elche (PI 57/2021) and it was prospectively registered to clinicaltrials.gov (NCT05390359).

### Instruments

The electrical characterization of the patellar tendon was performed using bioimpedance spectroscopy with the Keysight E4980AL precision LCR meter device (Keysight Technologies, California, USA). The utilized device, Keysight E4980AL, has a frequency range of 20 Hz–1 MHz and applies a sinusoidal alternating current test signal, with adjustable levels from approximately 1 µA to 20 mA. Also, the instrument has shown excellent accuracy of measurement (0.05%) according to the device manual. Although classical impedance representations and circuit models are described for interpretative purposes, no equivalent circuit fitting was performed here, and all analyses were based on directly measured impedance parameters across frequencies.

**Figure 2:**
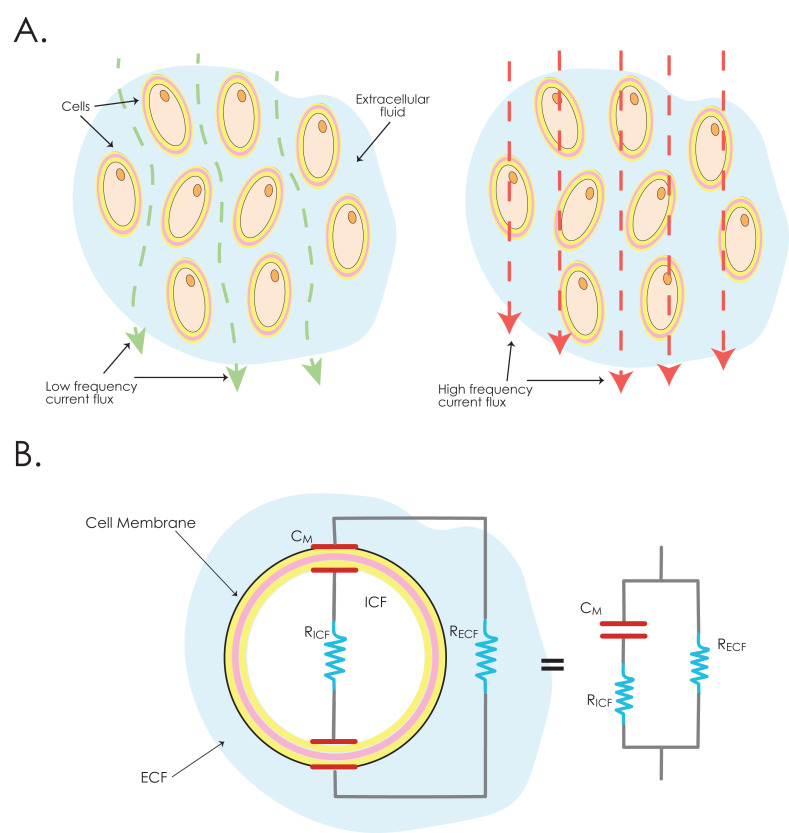
Electrical current conduction through biological tissues. (A) At low frequencies (green line), the current flows through the extracellular fluid. At higher frequencies (red line), the current traverses the cell membrane and intracellular fluid, apart from crossing the extracellular fluid between them. (B) Electrical impedance modeling of a biological cell, illustrating the *R*esistance of the *I*ntracellular *F*luid (R_ICF_) and *E*xtracellular *F*luid (R_ECF_). Note that the cell membrane behaves as a capacitor, generating *C*apacitance *M*embrane (C_M_), which accounts for the capacitive reactance in this arm of the circuit. This results in the tissue impedance to be modulated in frequency: this capacitor presents less impedance as the frequency of the current increases. The right side of the image displays the equivalent circuit. Adapted from 29.

The procedure involved the insertion of four monopolar needles: two needles at each pole of the patellar tendon (0.32 × 40 mm, steel material, IONCLINICS SL., Valencia, Spain). A tetrapolar configuration was used, with the outer needles delivering the alternating current and the inner needles measuring the resulting voltage drop across the tendon, allowing accurate intratendon impedance measurements, while minimizing electrode polarization effects (Aroom et al., 2009; Zhao et al., 2017). Assessments were performed at 201 frequencies, ranging from 3 KiloHertz (kHz) to 1 MegaHertz (MHz), using a measuring alternating current of 200 µA, a value within the safe and commonly used range for human tissues (Kyle et al., 2004). The current amplitude was set at 200 µA for safety and comfort and agreeing with other works in which currents of low intensity (<mA) are commonly used (Jaffrin & Morel, 2008; Sanchez et al., 2013). Additionally, this low intensity had the advantage of being below thresholds associated with tissue stimulation, heating, pain or discomfort perception. Using a wide range of frequencies allows characterizing the electrical properties of different tissular components of the tendon, differentiating both intracellular and extracellular aspects, due to the insulating properties of cell membranes (lipid bilayer) (Amini, Hisdal & Kalvøy, 2018; Bera, 2018) (Fig. 2A).

In bioimpedance spectroscopy, the measured variables are macroscopic electrical responses of a tissue segment to an externally applied alternating electric field. These variables do not represent intrinsic electrical properties of the biological material itself, but rather geometry-dependent parameters that reflect how ionic charge displacement occurs within the tissue under specific measurement conditions. More formally, electrical impedance (Z) is defined as the complex ratio between applied voltage and resulting current under an alternating electric field. Also, Bioimpedance quantifies the opposition encountered by the electrical current as it flows through a tissue, and it is conventionally defined by a cartesian equation (Aroom et al., 2009):

\begin{eqnarray*}Z=R+jX \end{eqnarray*}

where *R* represents the resistance, *X* the reactance and *j* the imaginary unit. Although electrical impedance (Z) is used here for simplicity, in bioimpedance spectroscopy impedance is frequency-dependent and should formally be expressed as Z(j*ω*), where *ω* represents the angular frequency. Thus, bioimpedance is a complex number derived from a real part (resistance) and an imaginary part (reactance) of the impedance:

-Resistance (*R*) represents the real component of impedance and reflects the opposition of the tissue to ionic conduction induced by an applied alternating electric field (units: Ω ohms). From a physiological perspective, lower R values indicate lower overall opposition to charge displacement within the measured tendon segment. Resistance shows limited frequency dependence compared to reactance and is mainly influenced by extracellular water content and matrix composition, providing indirect information on tissue hydration and structure (Yamaguchi et al., 2024).

-The Reactance (*X*) represents the imaginary component of impedance, and reflects the capacitive behavior of biological tissues, mainly associated with membrane polarization at cellular and subcellular interfaces (Ω). Reactance is inversely related to frequency (*f*) and capacitance (*C*) (*X* = 1/(2*πfC*)). At low frequencies, reactance is high due to the dominant influence of capacitive components. As frequency increases, Reactance reduces its contribution to the total impedance, defined by $ \left\vert Z \right\vert =\sqrt{{R}^{2}+{X}^{2}}$. Consequently, at low frequencies, impedance is predominantly determined by reactance, whereas at high frequencies, resistance becomes the principal contributor (Fig. 2B). This behavior highlights the frequency-dependent electrical response of biological tissues under alternating electric fields (Kyle et al., 2004). Reactance can be associated to tissue structure and integrity of cell membranes, inflammation, or edema, relevant in tendon pathology (Yoon et al., 2010).

**Figure 3:**
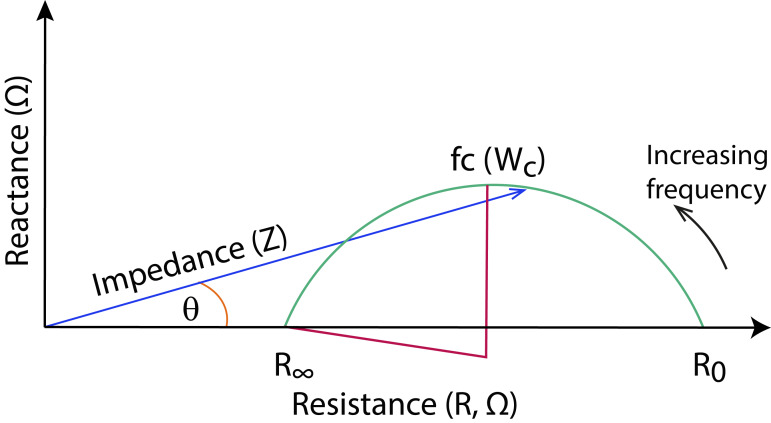
Graphically represented the components of bioimpedance in the same vector as a Cole-Cole plot, illustrating the imaginary part (Reactance, *y*-axis) and de real part (Resistance, *x*-axis). |*Z*| (Impedance), *θ* (Phase Angle), R_0_ (Resistance at zero frequency), R_∞_ (Resistance at infinite frequency), Wc (angular frequency at characteristic frequency). Modified of Ward & Brantlov (2023).

Alternatively, bioimpedance spectroscopy, expressed as Z(j*ω*), can also be represented by the polar form:

\begin{eqnarray*}Z(j\omega )=\mid Z\mid \angle \phi \end{eqnarray*}

where ∣*Z*∣ is the magnitude of the bioimpedance spectroscopy and *ϕ* is the phase angle. Referring the bioimpedance as a vector (Fig. 3).

-The Magnitude (∣*Z*∣) is the absolute value of the total impedance (*Z*), thus is always positive. The Magnitude is generally higher at low frequencies due to dominant capacitive effects and decreases as frequency increases. The magnitude, as a combination of resistance and reactance components, offers a simplified overview of the electrical behavior of a tissue (Kyle et al., 2004).

-The Phase Angle (*ϕ*) represents the time delay or phase shift between the voltage and current signals resulting from the interaction between resistive and reactive components of bioimpedance. Phase angle varies with tissue structure and frequency and is considered an indirect marker of cellular organization and membrane integrity. For instance, a fibrotic *versus* a highly structured tissue will show significant phase variations along frequencies. The Phase angle has been associated with tissue damage and structural alterations in musculoskeletal models (Nescolarde et al., 2013).

To facilitate interpretation, all the components of bioimpedance spectroscopy can be graphically represented in the same vector, using a Nyquist plot, where reactance is plotted against resistance across frequencies, according to the Cole-Cole model (Fig. 3).

Summarizing, this approach allows us to explore both extracellular and intracellular characteristics of tendon tissue, with each impedance parameter offering complementary insights. The parameters chosen (Resistance, Reactance, Magnitude and Phase Angle) are standard in the field and have been employed in prior musculoskeletal research for similar purposes (Jaffrin & Morel, 2008; Aroom et al., 2009; Scott et al., 2020; Yamaguchi et al., 2024).

Previous works studying bioimpedance changes on pathological tissues provide a guide on how to interpretate IT-BS assessments. For example, a collagenase-induced tendinitis in a rabbit model exhibits clear frequency-dependent alterations, including reduced resistive (100 Hz–10 MHz) and a lower dissipation factor (a composite parameter reflecting the resistance to reactance ratio) between 300 Hz and 3 MHz compared to healthy tissue (Yoon et al., 2010). This result is in accordance to works on muscle injury, where the resistance observed at 50 kHz frequencies decreases at the injury site, reflecting localized fluid accumulation. The same authors also find a reduction in Reactance and angle phase at 50 kHz, that could be interpreted as disruption of the cellular membrane integrity (Nescolarde et al., 2013). In this same direction, the decrease of reactance at 5, 50 and 250 kHz frequencies were correlated with an increase of biomarkers indicating exercise-induced muscle damage (EIMD) (Yamaguchi et al., 2024). Then, IT-BS changes can be related to specific pathological alterations in the tendon tissue: a reduction in resistance at low frequencies is suggestive of localized fluid accumulation (*i.e.,* inflammation) whereas a reduction in reactance and angle phase at low frequencies seem to relate to disruptions in the cellular membrane integrity.

Although 50 kHz has been used conventionally as a cutoff to differentiate extra and intracellular compartments (Ackmann, 1993; Gabriel, Gabriel & Corthout, 1996; Kyle et al., 2004), we decided to define the frequency domains (high and low frequency) based on the experimental characteristic frequency (ecf) of our sample. The ecf was defined as the frequency at which the absolute value of the reactance reached its maximum:

\begin{eqnarray*}\mathrm{fce}=f\text{such that}~{|}X(f){|}=max({|}X{|}). \end{eqnarray*}

This criterion corresponds to the frequency at which capacitive effects related to cell membranes are maximal and is commonly associated with the center of the beta dispersion. Using this approach, the experimental characteristic frequency of our basal measurements was established at 680.3 kHz.

### Procedures

**Figure 4:**
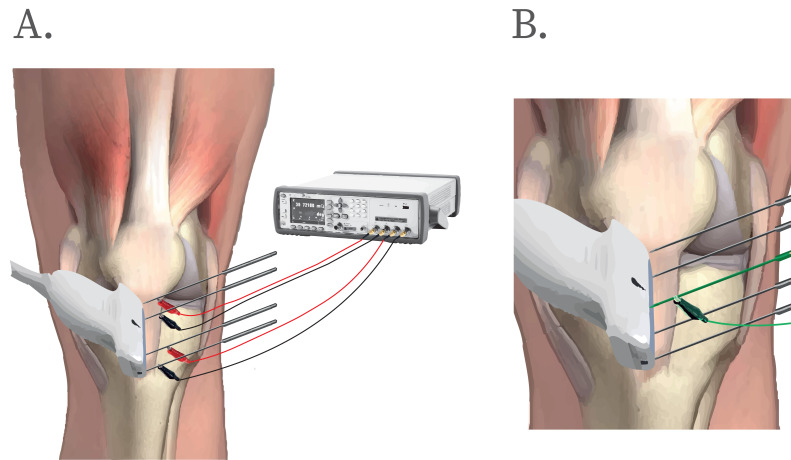
Procedure to measurement and PE application. (A) The needle was inserted into the patellar tendon under ultrasound guidance. The red lead functioned as the anode, while the white lead served as the cathode. (B) A needle was inserted at the midpoint of the patellar tendon (green needle), between the bioimpedance measurement needles, and connected to the device for electrical stimulation. Additionally, to evaluate the safety and potential adverse effects of bioimpedance measurements, pain perception from participants was monitored continuously during the procedure using a digital self-reported *V*isual *A*nalog *S*cale (VAS). The components of this figure (templates, graphics, and silhouettes) were generated with the assistance of ChatGPT (OpenAI).

In the first session, participants were positioned on a stretcher. Measurements were performed with participants in a supine position with the knee supported in approximately 30°  of flexion (0°  representing the complete anatomical extension), maintained passively using a triangular foam cushion. The skin of the target area was shaved and cleaned with alcohol-soaked gauze. The positions of the four needles (0.32 × 40 mm, steel material, IONCLINICS SL., Valencia) were determined by ultrasound and marked over the skin. The needle insertion points were determined by measuring the length of the patellar tendon *via* ultrasound. The four needles were inserted guided by ultrasound to ensure that the needle was inserted at the intended anatomical location and at the appropriate depth (the mid–deep portion of the tendon) and were positioned equidistant along the longitudinal axis to standardize inter-electrode spacing and ensure consistent current distribution through the tendon. Two needles were inserted proximally, near the inferior pole of the patella and two needles distally, adjacent to the tibial tuberosity. Once the needles were placed, they were connected to the Keysight E4980AL device *via* alligator clips (Fig. 4). In this tetrapolar configuration the outer needles delivered the alternating current, and the inner needles recorded the resulting voltage drop across the tendon to calculate the impedance of this tissue. The baseline measurement (test) was recorded, and a second measurement (retest) was performed five minutes later to evaluate intra-session reliability. The needles were maintained in the same location during the intra-session assessments.

In the second session, the procedure was identical to the baseline measurement of the first session. Following this, a single session of PE was applied. First, an additional needle used for PE application was inserted into the center of the patellar tendon, guided by ultrasound. Bioimpedance was measured after needle insertion as a control measure, in order to control for potential bioimpedance alterations produced by the needle itself (De la Cruz Torres et al., 2016). Thereafter, PE was applied: 3 square pulses of high-intensity galvanic current (three mA), each lasting for 3 s. The pulses were separated by 3 s rest intervals, with a 1 s ramp-up period preceding each pulse (Moreno et al., 2017). The total duration of the protocol was 18 s and the stimulation was applied with a certified medical device (EPTE 2.0 Bipolar System, Ionclinics) (Fig. 4). The needle served as the cathode, while the circuit was completed using an electrode placed on the medial surface of the tibia. Fifteen minutes later, another bioimpedance measurement was conducted to assess the changes produced by PE, with the PE needle still in place.

Finally, the adverse effects of IT-BIS were mainly assessed through digital and continuous VAS during the measurement (0–100), which participants manipulated in real time *via* a touchscreen slider during the bioimpedance measurement to continuously rate their perceived pain.

### Statistical analysis

Prior to statistical analysis, an objective and reproducible outlier detection procedure was applied. The root mean square error (RMSE) between each subject’s individual trace and the corresponding group mean trace was computed across all measured frequencies, providing a global measure of deviation that accounts for the entire frequency-dependent behavior. Outliers were identified using Tukey’s criterion on the RMSE distribution, defined as outliers when the RMSE exceeded the third quartile plus 1.5 times the interquartile range (Q3 + 1.5*IQR). Outliers were excluded from subsequent analyses for the corresponding variable. This approach allowed the identification of globally atypical spectra in a transparent and data-driven manner, while preserving the expected inter-individual variability of bioimpedance measurements.

Participant characteristics and bioimpedance parameters are presented as mean ± Standard Deviation (SD) or percentages, as appropriate. For all analysis, SPSS (version 28; SPSS Inc, Chicago, IL) was used. Variables were checked for normality with the Kolmogorov–Smirnov test and homogeneity of variances with Levene’s test.

First, for the analysis of the bioimpedance intra- and inter- session reliability, we calculated indexes of relative (Intra-class Correlation Coefficient: ICC_1,1_) and absolute reliability (Standard Error of Measurement: SEM. Minimal Detectable Change: MDC) (Atkinson & Nevill, 2012; Koo & Li, 2016). Relative reliability was conceptualized as the degree to which individuals maintain their position in a sample with repeated measurements, and absolute reliability as the degree to which repeated measurements vary for individuals (Medina-Mirapeix et al., 2019). The reliability was classified as excellent (ICC >0.90), good (ICC = 0.76–0.90), moderate (ICC = 0.51–0.75), and poor (ICC < 0.50) (Koo & Li, 2016). ICC was calculated as (*MSR* − *MSE*)/ $(MSR+ \left( k-1 \right) \ast MSE+ \frac{k}{n} \ast \left( MSc-MSE \right) )$, where MSR = mean square for rows; MSE = mean square for error; MSC = mean square for columns; n = number of subjects; k = number of raters/measurements (Koo & Li, 2016). MDC was calculated for the 95% CI as $MDC95=SEM\times 1.96\times \sqrt{2}$, where $SEM=\mathrm{SD}\sqrt{(1-\mathrm{ICC})}$.

Second, the sensitivity to change was conceptualized as the tool’s ability to measure the change over a specified time frame using a previously described procedure (Husted et al., 2000). The sensitivity to change was determined using a paired *t*-test to determine whether the mean difference between measurements was statistically significant, and supplemented with two effect-size metrics as recommended by Husted et al. (2000) and similar to what was carried out by other studies (Navarro-Pujalte et al., 2018; Martín-San Agustín et al., 2020; Sánchez Martínez et al., 2024). First, the Standardized Response Mean (SRM) was calculated as the mean change divided by the standard deviation of the change scores, providing an estimate of the magnitude of change that is independent of sample size. Second, Cohen’s *d* was calculated based on the mean difference and pooled standard deviation and interpreted according to conventional thresholds (*d* < 0.2: trivial, 0.2–0.5: small, 0.5–0.8: medium, and >0.8: large). Confidence intervals were calculated assuming the change score were normally distributed (Navarro-Pujalte et al., 2018). Values of 0.20, 0.50, and 0.80 or higher have been proposed in the literature (Husted et al., 2000) to represent small, moderate, and large change magnitudes, respectively.

The sample size was calculated using the formula for reliability studies based on Confidence Intervals (CIs) described by Streiner & Norman (2008). With the number of tests (k) equal to 2, the CI around r (the reliability coefficient) of 0.05, and an estimated r of 0.95, the sample size (n) was calculated as a minimum of 25 participants. The expected reliability coefficient (*r* = 0.95) was selected based on the exploratory nature of the study and the controlled measurement conditions (a single experienced examiner, fixed electrode position during intrasession assessments), in which excellent short-term reliability was anticipated. The sample size was increased to account for measurement errors in the measuring device (Mokkink et al., 2022). The raw measurements are available in the Supplemental Files 1 and  2.

## Results

A total of 40 participants were initially recruited and completed all assessments. Following the application of predefined signal-quality criteria, 27 participants were retained for the final analysis. The final sample had a mean age of 25.2 ± 3.4 years (11 females and 16 males), reported a physical activity level of 4,150.7 ± 2,657.2 mets/week and a score of 96.4 ±  5.2 of VISA-P.

**Figure 5:**
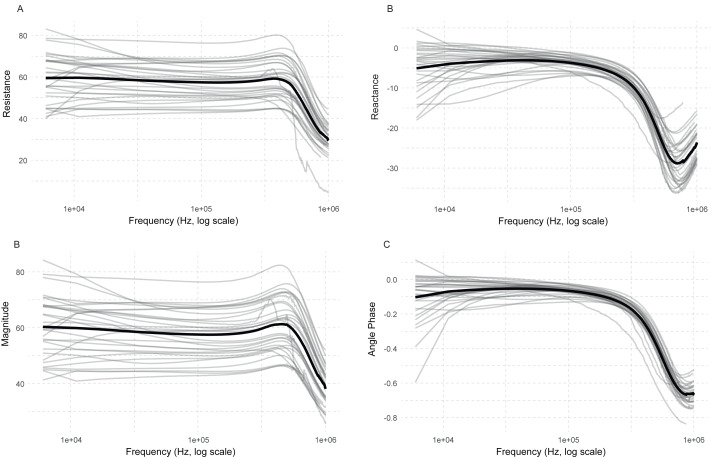
Bioimpedance plots showing mean (black) and individual subject traces (grey) under basal conditions across the frequency spectrum. The *x*-axis is displayed on a logarithmic scale.

The mean trace and the individual traces of all subjects across frequencies for resistance, reactance, magnitude, and phase angle are plotted in Fig. 5. The ecf was calculated as 680 kHz. The results calculated by low frequency included an average of 135 frequencies, ranging from 5,000 kHz to 680 kHz. Meanwhile, the high-frequency results involved the average of 55 frequencies, from 690 kHz to 1 MHz.

### Intra- and inter- session reliability

First, we analyzed the intra-session reliability of the bioimpedance assessment with electrodes maintained in a fixed position. The data and statistics are summarized in Table 1. ICC were interpreted according to the thresholds proposed by Koo & Li (2016): values below 0.5 were considered poor, 0.5–0.75 moderate, 0.75–0.9 good, and above 0.9 excellent. Under these conditions, the analysis revealed excellent relative reliability across all parameters in both the range of high and low frequency. Specifically, resistance demonstrated ICC of 0.998 in the low frequency and 0.927 in the high frequency; reactance showed values of 0.987 and 0.997, respectively; magnitude reached 0.999 and 0.962; and phase angle yielded ICCs of 0.979 and 0.999, respectively. Regarding the absolute reliability analysis, the high frequency parameters showed a SEM < 6.5% (range 0.01% to 2.14%) while the low frequency parameters showed SEMs from 0.01% to 2.14%. Consequently, when electrode position is maintained, the intra-session reliability can be considered excellent in terms of both relative and absolute measures, with comparable performance across low- and high-frequency parameters.

**Table 1:** Intra-session reliability of bioimpedance assessment for electrical characterization of patellar tendon.

Parameter	**Frequency**	**Test (SD)/Retest (SD)**	**ICC**	**SEM (%)**	**MDC (%)**
Resistance (Ω)	Low	56.68 (8.99)/56.36 (9.05)	0.998 (0.997–0.999)	0.40 (0.74)	1.12 (2.05)
	High	35.20 (6.86)/35.83/7.18)	0.927 (0.846–0.966)	2.14 (6.41)	5.93 (17.76)
Reactance (Ω)	Low	−13.86 (2.39)/−13.79 (2.58)	0.987 (0.971–0.994)	0.28 (1.94)	0.79 (5.36)
	High	−27.01 (4.37)/−27.13 (4.39)	0.997 (0.993–0.999)	0.24 (0.89)	0.67 (2.47)
Magnitude	Low	58.33 (8.28)/ 59.10 (9.11)	0.999 (0.998–1)	0.29 (0.5)	0.80 (1.38)
	High	44.67 (7.42)/45.33 (7.63)	0.962 (0.919–0.983)	1.52 (3.51)	4.22 (9.73)
Phase angle (rad)	Low	−0.24 (0.05)/−0.24 (0.04)	0.979 (0.953–0.990)	0.01 (2.18)	0.02 (6.04)
	High	−0.65 (0.06)/−0.65 (0.06)	0.999 (0.997–0.999)	0.01 (0.29)	0.01 (0.81)

**Notes.**

SD Standard Deviation ICC Intraclass Correlation Coefficient SEM Standard Error of Measurement MDC Minimal Detectable Change

Second, we analyzed inter-session reliability. The data and statistics are summarized in Table 2. This analysis showed poor reliability for almost all parameters at both high- and low- frequencies. Only the phase angle at low frequency demonstrated moderate reliability (ICC = 0.53). The remaining parameters showed poor reliability, with ICC values ranging from 0.09 to 0.39 and confidence intervals often extending to zero or negative values. While the absolute reliability analysis for the high frequency parameters showed a SEM < 19.5% (range 7.29% to 19.01%), the low frequency parameters showed SEMs < 18% (range 10.81% to 18%). Thus, the analysis showed a limited inter-session reliability in both frequencies range.

**Table 2:** Inter-session reliability of bioimpedance assessment for electrical characterization of patellar tendon.

Parameter	**Frequency**	**Test (SD)**	**ICC**	**SEM (%)**	**MDC (%)**
Resistance (Ω)	Low	51.39 (10.77)	0.157 (−0.255–0.521)	9.35 (18)	25.9 (49.88)
	High	34.64 (5.62)	0.200 (−0.222–0.559)	6.53 (19.01)	18.1 (52.7)
Reactance (Ω)	Low	−12.96 (2.43)	0.315 (−0.103–0.638)	2.03 (14.82)	5.63 (41.07)
	High	−26.23 (4.97)	0.244 (−0.188–0.597)	3.93 (15.12)	10.9 (41.9)
Magnitude	Low	55.91 (7.20)	0.330 (−0.97–0.654)	6.91 (12.63)	19.15 (35)
	High	43.67 (6.59)	0.390 (−0.28–0.692)	5.66 (13.08)	15.68 (36.25)
Phase angle (rad)	Low	−0.24 (0.05)	0.528 (0.157–0.768)	0.03 (10.82)	0.08 (29.99)
	High	−0.63 (0.04)	0.087 (−0.329–0.475)	0.05 (7.29)	0.13 (20.21)

**Notes.**

SD Standard Deviation ICC Intraclass Correlation Coefficient SEM Standard Error of Measurement MDC Minimal Detectable Change

### Sensitivity to change

To explore the sensitivity to change, a PE intervention session was applied. As PE intervention implies a needle insertion, first, to characterize the effect of the needle insertion in the IT-BIS measurement, we explored the sensitivity to change of each bioimpedance parameter. Table 3 presents the sensitivity to detect a change produced by the presence of an intratendon needle through bioimpedance. The needle insertion significantly reduced all parameters at low frequency with the pre- post differences analysis. Specifically, we found changes at resistance (*p* = 0.009; SRM = −0.54), reactance (*p* = 0.004; SRM −0.61), magnitude (*p* = 0.039; SRM = −0.4), and phase angle (*p* = 0.02; SRM = −0.67). Curiously, the high frequency parameters did not significantly change with the needle insertion.

**Table 3:** Sensitivity to change of bioimpedance assessment for electrical characterization of patellar tendon with the presence of an intratendon needle.

**Parameter**	**Frequency**	**Difference pre-post needly**	**Paired** ***t*** **-test**	**SRM (95% CI)**	**Cohen’s** ***d*** ** (95% CI)**
Resistance (Ω)	Low	1.52 (2.84)	0.009	−0.54 (−0.8588 to −0.2117)	0.54 (0.09 to 0.97)
	High	0.52 (3.49)	0.264	−0.15 (−0.5552 to 0.4197)	0.15 (−0.31 to 0.60)
Reactance (Ω)	Low	1.28 (2.11)	0.004	−0.61 (−0.9468 to −0.2729)	0.61 (0.16 to 1.05)
	High	−0.49 (1.66)	0.101	0.29 (−0.2538 to 0.6233)	0.30 (−0.16 to 0.74)
Magnitude ** (Ω)**	Low	0.92 (2.29)	0.039	−0.40 (−0.7204 to −0.05401)	0.40 (−0.05 to 0.85)
	High	0.55 (3.74)	0.270	−0.15 (−0.5412 to 0.4366)	0.15 (−0.32 to 0.61)
Phase angle (rad)	Low	0.04 (0.06)	0.002	−0.67 (−1.0270 to −0.3449)	0.67 (0.21 to 1.11)
	High	−0.01 (0.03)	0.194	0.20 (−0.2979 to 0.5633)	0.20 (−0.25 to 0.64)

**Notes.**

SRM Standardized Response Mean CI Confidence Interval

The units were Ω for Resistance, reactance and magnitude and radians for Phase angle.

Next, Table 4 presents the sensitivity to detect a change produced by PE through bioimpedance. Statistically significant pre–post differences were found exclusively at high frequency, affecting resistance (*p* = 0.001; SRM = 0.67), magnitude (*p* = 0.006; SRM = 0.54), and phase angle (*p* = 0.003; SRM = 0.60).

**Table 4:** Sensitivity to change of bioimpedance assessment for electrical characterization of patellar tendon after percutaneous electrolysis.

**Parameter**	**Frequency**	**Difference pre-post electrolysis**	**Paired** ***t*** **-test**	**SRM (95% CI)**	**Cohen’s** ***d*** ** (95% CI)**
Resistance (Ω)	Low	−1.61 (6.50)	0.124	0.25 (−0.19 to 0.57)	0.25 (−0.17 to 0.66)
	High	−2.61 (3.81)	0.002	0.68 (0.05 to 1.25)	0.68 (−0.21 to −1.14)
Reactance (Ω)	Low	−0.28 (1.89)	0.237	0.15 (−0.29 to 0.81)	0.15 (−0.25 to 0.55)
	High	−0.86 (2.19)	0.037	0.39 (−0.19 to 1.04)	0.49 (−0.04 to 0.81)
Magnitude (Ω)	Low	−0.32 (1.58)	0.181	0.20 (−0.32 to 0.67)	0.21 (−0.23 to 0.63)
	High	−1.74 (3.14)	0.010	0.55 (0.22 to 0.89)	0.55 (−0.09 to 1.01)
Phase angle (rad)	Low	−0.01 (0.05)	0.352	0.08 (−0.36 to 0.75)	0.08 (−0.33 to 0.49)
	High	−0.05 (0.08)	0.006	0.57 (−0.15 to 2.37)	0.57 (−0.12 to 1.01)

**Notes.**

SRM Standardized Response Mean CI Confidence Interval

The units were Ω for Resistance, reactance and magnitude and radians for Phase angle.

Taken together, these findings indicate a frequency-dependent response. Needle insertion induced significant changes only at low frequency, consistent with predominantly extracellular alterations. In contrast, PE elicited significant changes exclusively at high frequency, suggesting a greater involvement of intracellular components. This differential pattern supports the potential of IT-BIS to discriminate between extracellular and intracellular tissue modifications.

### Adverse effects of IT-BIS

The final sample size for this analysis was 27 participants. During the first and second session basal measurements, 26 subjects scored 0 pain intensity. Only one participant per session reported any pain, with mean and maximum values of 0.15 and 13 on the first day and 0.48 and 41 on the second day, respectively. A similar pattern was observed during the retest measurements. Only one subject reported pain in each retest session. On the first retest day, the mean pain score was 7.36, with a maximum of 14. On the second day, the mean was 0, and the highest value was 13. Additionally, two participants reported maximum scores of 14 and 13 out of 100 following needle insertion during the second session. Therefore, a minimal level of pain was reported five times across 108 measurements (27 *subjects*  ×  4 *assessments*) corresponding to an adverse effect occurrence rate of 4.63%. These instances were transient, and no other adverse effects were reported by the participants, indicating that IT-BIS is a safe and low-risk procedure.

## Discussion

The purpose of this study was to explore the electrical characterization of a tendon using IT-BIS and to investigate the reliability, sensitivity to change and safety of this minimally invasive procedure. Overall, the technique showed excellent short-term measurement stability when the electrodes were maintained in place and was sensitive to changes induced by a PE intervention. However, its inter-session reliability was poor to moderate.

The intrasession reliability obtained in the bioimpedance assessment proved to be excellent for high and low frequency parameters. To our knowledge, this is the first study assessing the reliability of IT-BIS, so it is not possible to compare our results with others using the same assessment technique. While other assessment methods of tendon as intratendinous signal using MRI or ultrasound tissue characterization (UTC) also showed excellent reliability (Shalabi et al., 2005; Van Ark et al., 2019), the macrostructural tissular characteristic evaluated by these methods are critically different from what is evaluated by IT-BIS. Thus, there is room for combining these assessment methods for a multidimensional tendon properties assessment. Additionally, the relationship between these outcomes and the clinical features associated with pathological states involving the tendon should be included to decide on the usefulness of this assessment method. Due to the maintenance of needles during the intrasession measurements, the reliability results obtain could only be applied to clinical contexts in which intrasession procedures were assessed.

The intersession reliability was shown to be poor to moderate for the bioimpedance assessment. This is partially expected due to the procedural variation of the placement of the needle electrodes between the first and second sessions. Moreover, variations of the hydration status of the participants could reduce the intersession reliability, especially at low frequencies as this parameter strongly influences the BIS measurement (Birgersson et al., 2013).

PE intervention significantly reduced the resistance, reactance, magnitude and phase angle specifically at high frequencies, demonstrating that IT-BIS is sensitive to change. We acknowledge that the maintenance of the needle inserted in the tendon after the PE intervention could influence the analysis. However, the IT-BIS control measurement after the needle insertion, before PE application, allow us to suggest that IT-BIS is influenced by maintaining the needle inserted. The PE intervention involves a galvanic current circulating through the tendon, as well as a needle (used as electrode to deliver the current). The modification of IT-BIS parameters by PE were at high frequency could provide a change on the state of the less hydrated components of the tendon, such as the collagen extracellular matrix. In accordance with this hypothesis, two recent studies reported that PE acutely alters collagen expression and structure in the Achilles tendon of mice and rats (Sánchez-Sánchez et al., 2020; Peñin Franch et al., 2022). These results can be aligned with the reduction on the resistance of a collagenase-induced tendinitis in a rabbit model on the range of 100 Hz–10 MHz (Yoon et al., 2010).

Despite being minimally invasive, adverse effects associated with IT-BIS presented a low incidence rate and pain was reported in isolated cases and minimal intensity levels, supporting the conclusion that IT-BIS is a safe procedure. It is relevant to mention that IT-BIS was still not painful even after applying PE, which is a painful procedure that induces a proinflammatory state (Sánchez-Sánchez et al., 2020). This suggests that this measure is probably pain free even in cases of tendons displaying increased pain sensitivity, such as tendinopathies.

From our point of view and taking into account the results, IT-BIS as it is executed here would be useful to characterize microscopic alterations in the tendon structures produced by acute interventions, such as the PE applied here or mechanical loading should be focused in two: its high intrasession reliability and sensitivity to change will permit to determine whether the electrical characteristics of the tendon have varied acutely. But of course, it will be ideal to improve the intersession reliability of IT-BIS to make it a valid tool to follow changes occurring in longer time spans, such as tendon adaptation to chronic mechanical loading, structural degeneration in tendinopathy or tendon repair after surgery for ruptures. For this purpose, future studies should further standardize the measurement procedure, particularly by controlling to some extent the level of hydration of the patients: this can be achieved by systematizing the hour of the measurement and regulating water consumption in the hours prior to measurement. Moreover, the depth of the needle insertion into the tendon should be controlled.

### Limitations

Some limitations should be considered when interpretating this study. First, only intratester reliability analysis was conducted. Since the technique requires training and experience, it would be advisable to evaluate the interrater reliability in the future. Second, it is important to remember that the needles were located at the same location during the intra-session assessments, which could result in an overestimation of the reliability and limits the clinical transference. Future studies that deep further in IT-BIS reliability could extract and introduce the needles to explore this factor specifically. Third, only two measurements were obtained per session, offering a limited estimate of intra-session variability. Future IT-BIS reliability studies should include three or more repeated measurements per session to enable a more robust characterization of measurement stability. Fourth, the lack of examiner blinding increased the risk of bias. Although the IT-BIS assessment and standardized electrode positioning procedures were intended to minimize potential operator-related bias, the lack of blinding could have influenced intra- and inter-session reliability. Another limitation was that the two sessions were conducted at different times of the day, potentially influencing the condition of the tendon tissue: this factor could have contributed to the poor inter-session reliability observed, as tissue hydration is influenced by the circadian rhythm (Camilion et al., 2022; Dudek et al., 2023). Also, we did not systematically control or quantify circadian-related hydration changes. This factor should be considered in future reliability studies. Finally, PE was used as a technique to induce changes in the tendon and assess the sensitivity to bioimpedance change, however the present study does not provide direct evidence of microscopic structural alterations, as no histological or microscopic examinations were performed in parallel with the IT-BIS measurements. Although PE evidence supports that could induce molecular and microscopic anatomical changes in the tendon, such as an increase of the expression of proinflammatory factors or collagen reorganization (for a review on the biological effects of PE see Sánchez-Sánchez et al., 2020), the effect size of this intervention over the electrical characteristics of the tendon is not known. Therefore, it would be advisable to study the changes produced in the tendon impedance after the application of techniques with specific and well-known physiological effects, such as degradation of the collagen of the extracellular matrix by collagenase injection (Sánchez-Sánchez et al., 2020).

## Conclusions

IT-BIS is a safe, minimally invasive, painless and sensitive technique that can detect microscopic alterations in the tendon structure through electrical characterization at different frequencies. Its reliability at the intrasession level ranges is excellent, although its intersession reliability is poor to moderate. Therefore, IT-BIS is useful to characterize immediate changes produced in the tendon, or when normalization is possible towards a baseline measurement. Future studies should standardize the IT-BIS assessment procedure to improve its intersession reliability, as well as to assess its clinical utility applying it to characterize pathological tendons.

##  Supplemental Information

10.7717/peerj.21084/supp-1 Supplemental Information 1 Dataset of measurrements of bioimpedance reliability

10.7717/peerj.21084/supp-2 Supplemental Information 2 Codebook

10.7717/peerj.21084/supp-3 Supplemental Information 3 STROBE statement

## Additional Information and Declarations

### Data Availability

The following information was supplied regarding data availability: The raw data and code are available in the Supplemental Files.
