# Supplementary material for: Intratendon bioimpedance spectroscopy: a first step exploring reliability and sensitivity to change
Source: PeerJ. 2026 May 21;14:e21084. doi: 10.7717/peerj.21084 (PMC13198849; doi:10.7717/peerj.21084)
Supplement: Supplemental Information 2 [file peerj-14-21084-s002.docx]

**Descriptive measures**

Variable “Subject”: The codification of each subject

Variable “Included_final_analysis”: The codification of the subject included in the final analysis

- No: The subject was NO included in the final analysis
- Yes: The subject was included in the final analysis

Variable “Sex”:

1. Male
2. Female

“Age”: The age of each subject in years

“VISA-P and IPAQ”: The total score of each scale, respectively.

**Outcome measures**

The four outcome measures are Resistance, Reactancy, Magnitude and Phase (Angle Phase). For each variable has been used the same name codification to the different conditions and measures. We are to explain the codification to name “Variable”, which can be replaced by Resistance, Reactancy, Magnitude and Phase:

Variable description for intra-session reliability:

- “Variable_day1_low_pre_test”: Variable measured during session at day 1 at low frequency range during pre-test.
- “Variable_day1_low_post_test”: Variable measured during session at day 1 at low frequency range during post-test.
- “Variable_day1_high_pre_test” and “Variable_day1_low_post_test”:”: The same variable described above pre and post-test, but for high frequency.

Variable description for inter-session reliability and sensitivity to change:

- “Variable_day2_low_pre_test”: Variable measured during session at day 2 at low frequency range during pre-test. To compare with measurements of day1 pre-test.
- “Variable_day2_low_postneedle”: Variable measured during session at day 2 at low frequency range after needle insertion.
- “Variable_day2_low_postPE”: Variable measured during session at day 2 at low frequency range after percutaneous electrolysis treatment
